# Unveiling the role of O(6)-methylguanine-DNA methyltransferase in cancer therapy: insights into alkylators, pharmacogenomics, and others

**DOI:** 10.3389/fonc.2024.1424797

**Published:** 2024-07-11

**Authors:** Lizhen Chen, Alex Wen

**Affiliations:** ^1^ Department of Pharmacy, The First Hospital of Putian City, Putian, Fujian, China; ^2^ Dana-Farber Cancer Institute, Harvard Medical School, Boston, MA, United States; ^3^ Faculty of Synapse Program, Martinos Center, Massachusetts General Hospital, Harvard Medical School, Boston, MA, United States

**Keywords:** O(6)-methylguanine-DNA methyltransferase (MGMT), alkylator, cancer chemotherapy, DNA repair, pharmacogenomics

## Abstract

Cancer chemotherapy is advancing as we understand how cellular mechanisms and drugs interact, particularly involving the enzyme MGMT, which repairs DNA damage that can cause cancer. This review examines MGMT’s role in DNA repair, its impact on chemotherapy, and its complex interaction with radiation therapy. MGMT activity can both protect against mutations and cause drug resistance. Modulating MGMT could improve treatment efficacy and tailoring therapy to MGMT status may enhance patient outcomes. Understanding MGMT is crucial for developing precise cancer treatments and advancing patient care.

## Introduction

In the evolving landscape of cancer chemotherapy, the dynamic interactions between endogenous cellular components and exogenous therapeutic agents are progressively being elucidated, offering promising prospects for the development of impactful therapeutic interventions. Within this complex interplay lies O(6)-methylguanine-DNA methyltransferase (also known as methylguanine methyltransferase, MGMT), an enzyme of paramount importance that orchestrates the repair of DNA damage (1), particularly the methylation of the O-6 position of guanine—a modification that can lead to functional detriments.

The significance of MGMT extends beyond its reparative functions; it also engages in a nuanced relationship with a class of chemotherapeutic drugs known as alkylating agents, which include temozolomide and dacarbazine, among others (2–4). These agents exert their antineoplastic effects by introducing alkyl groups into the DNA structure, thereby disrupting the replication and transcription processes essential for cancer cell proliferation. However, the efficacy of these drugs can be markedly influenced by the activity of MGMT, which can remove these alkyl groups and, in doing so, confer resistance to the therapeutic effects of the alkylating agents. Furthermore, MGMT seems to interplay with radiation therapy and adds another layer of complexity to its role in cancer treatment (5–9).

This article aims to provide a comprehensive exploration of the multifaceted role of MGMT in the context of cancer therapy, examining its critical function in DNA repair, its impact on the effectiveness of alkylating chemotherapeutic agents, and its potential modulation of the outcomes of radiation.

## Pharmacogenomic rationale of MGMT

MGMT is an essential DNA repair enzyme that plays a crucial role in rectifying the cellular damage induced by alkylating agents. These agents are a class of chemotherapeutic drugs that exert their cytotoxic effects by transferring alkyl groups to the DNA helix through covalent bonding, leading to significant DNA damage that can result in cell apoptosis or necrosis. The MGMT enzyme reverses in this process by removing the alkyl groups, thereby preventing the formation of DNA adducts that can compromise genomic integrity and contribute to carcinogenesis. The activity of MGMT is, therefore, a critical factor in the cell response to alkylating chemotherapy, as it can influence the effectiveness of the treatment and the survival of the cancer cells.

The genetic underpinnings of O-6-methylguanine DNA methyltransferase are rooted in the MGMT gene, which is located on chromosome 10q26. This gene features a promoter region that is rich in CpG dinucleotides, commonly referred to as CpG islands. These islands are key regulatory elements that control gene expression through epigenetic mechanisms, one of which is methylation. Methylation of the CpG islands within the MGMT promoter region is a significant epigenetic modification that plays a pivotal role in downregulating MGMT expression. A high degree of CpG methylation correlates with the silencing of MGMT gene expression, which, in turn, predicts a reduced level of the MGMT enzyme within the cell. This reduction has profound implications for the sensitivity of cancer cells to alkylating chemotherapy, as lower levels of MGMT are associated with an increased susceptibility to the cytotoxic effects of these drugs. Understanding the methylation status of the MGMT gene can, therefore, provide valuable prognostic information and guide the selection of therapeutic strategies in the treatment of various cancers (2).

The ‘Stupp trial’ and the RTOG0525 trial have provided definitive evidence for the prognostic significance of MGMT gene promoter methylation in patients newly diagnosed with glioblastoma multiforme (GBM). The methylation status of the MGMT gene has been identified as a potent prognostic indicator, with data revealing a median survival duration of 21.2 months in patients whose GBMs exhibit MGMT promoter methylation, in stark contrast to a median survival of only 14.0 months in cases where the MGMT gene is unmethylated. These findings underscore the importance of MGMT status as an indicating factor of therapeutic outcomes and highlight the potential for tailoring treatment strategies based on the methylation profile of the MGMT gene in GBM (10).

The repair function of MGMT is for maintaining the integrity of cellular processes through its reversing DNA alkylation. This enzymatic activity, while protective in normal cells, paradoxically contributes to the development of resistance against chemotherapeutic alkylating agents within cancerous cells. Alkylating agents such as temozolomide and dacarbazine are integral components in the therapeutic regimens for various malignancies, including brain tumors, sarcomas, and lymphomas. However, the therapeutic efficacy of these drugs is often undermined by the active presence of MGMT within the tumor cells, which effectively repairs the very DNA damage these chemotherapeutic agents are designed to inflict.

The predictive value of MGMT methylation status as a biomarker was notably corroborated by the Stupp trial, which demonstrated a substantial survival advantage when temozolomide was administered in conjunction with standard radiotherapy for patients with MGMT-methylated glioblastoma, as opposed to those with an unmethylated MGMT gene. This pivotal study provided compelling evidence of the enhanced sensitivity to temozolomide in the presence of MGMT promoter methylation (11). Further investigations have consistently reinforced the enduring relevance of MGMT gene promoter methylation as a robust predictor of responsiveness to temozolomide. The methylation status of the MGMT promoter in glioblastoma serves not only as a prognostic indicator of disease outcome but also as a predictive marker for the efficacy of treatment with alkylating agents, thereby informing clinical decision-making and personalized treatment strategies (12).

## Relevance of MGMT methylation across cancer types

### Colorectal cancer

Studies have demonstrated that MGMT methylation plays a significant role in colorectal cancer, influencing tumor sensitivity to alkylating agents (13, 14). Wenger and Carén (15) highlighted the diagnostic value of methylation profiling in diffuse gliomas, noting its detection in colorectal cancer, suggesting a potential avenue for therapeutic intervention.

### Small cell lung cancer

Although less explored, the presence of MGMT methylation in small cell lung cancer indicates a similar mechanism of chemoresistance as observed in gliomas. The exploration of MGMT methylation status in this context could pave the way for personalized treatment strategies, enhancing the efficacy of chemotherapy regimens (16).

### IDH mutant astrocytoma and oligodendroglioma

The relevance of MGMT methylation in IDH mutant astrocytomas and oligodendrogliomas presents a complex landscape (17). While GBM shows a clear correlation between MGMT methylation and therapeutic response, these other glial tumors exhibit a nuanced interaction. Aquilanti et al. (18) provided updates on prognostic markers for gliomas, emphasizing the prognostic importance of MGMT methylation in low-grade gliomas, including astrocytomas and oligodendrogliomas. Furthermore, Gupta and Salunke (19) discussed molecular markers of glioma, underscoring the need to distinguish oligodendrogliomas based on MGMT promoter methylation status, which may offer insights into tumor behavior and response to therapy.

The exploration of MGMT methylation across various cancer types underscores its potential as a biomarker for predicting treatment response and tailoring therapeutic approaches. The evidence suggests that beyond GBM, colorectal cancer, small cell lung cancer, IDH mutant astrocytoma, and oligodendroglioma could benefit from a deeper understanding of MGMT methylation dynamics.

## Temozolomide/dacarbazine/streptozocin

### Temozolomide

**Temozolomide,** an orally administered alkylating chemotherapeutic agent, is widely utilized in the clinical management of various malignancies, including glioblastoma, neuroendocrine tumors, melanoma, and sarcoma. It is also prescribed off-label for the treatment of central nervous system metastases originating from solid tumors. The therapeutic efficacy of temozolomide is significantly influenced by the intracellular levels of MGMT, an enzyme that plays a crucial role in the repair of DNA damage caused by alkylating agents. Glioblastoma cells exhibiting elevated MGMT expression are capable of repairing the alkylation-induced DNA lesions, thereby diminishing their sensitivity to the cytotoxic effects of temozolomide. Consequently, even after an initial therapeutic response, resistance to temozolomide frequently develops, with MGMT being implicated as a principal factor in the emergence of this resistance.

Continued research at the molecular level has elucidated that the epigenetic modification of the MGMT gene, specifically the hypermethylation of its promoter region, is instrumental in augmenting the sensitivity of tumor cells to alkylating agents. The hypermethylation of the MGMT promoter leads to transcriptional silencing of the gene, resulting in reduced synthesis of the MGMT enzyme. Clinical observations have substantiated that patients with glioblastoma characterized by a hypermethylated MGMT promoter exhibit a favorable response to temozolomide therapy. In contrast, patients whose tumors possess a hypomethylated MGMT promoter do not experience the same level of therapeutic benefit (11).

In light of these findings, the National Comprehensive Cancer Network (NCCN) has underscored the importance of evaluating MGMT promoter methylation status as a critical component of the molecular diagnostic workup for all high-grade gliomas, encompassing both grade 3 and grade 4 tumors. This assessment is not only prognostic but also informs therapeutic decision-making, guiding the selection of treatment regimens that are more likely to yield positive clinical outcomes for patients afflicted with these aggressive brain tumors (12).

### Dacarbazine

**Dacarbazine,** an alkylating chemotherapeutic agent, is conventionally utilized in the management of melanoma and Hodgkin’s lymphoma. However, its clinical efficacy is frequently compromised by resistance mediated through the DNA repair enzyme MGMT. The mechanism of dacarbazine involves the alkylation of DNA at the O(6) and N(7) positions of guanine, which can result in the formation of DNA double-strand breaks, a form of cytotoxic damage that impedes cancer cell proliferation. Analogous to the mode of action of temozolomide, dacarbazine exerts its antineoplastic effects by inducing DNA alkylation, and the therapeutic potential of dacarbazine is attenuated by the MGMT enzyme, which can restore the integrity of DNA by reversing the alkylation damage (20).

The metabolic pathway of dacarbazine is characterized by its biotransformation into the active metabolite 5-(3-methyl-triazen-1-yl)-imidazole-4-carboxamide (MTIC), a process facilitated by the cytochrome P450 enzymatic system. It is noteworthy that both dacarbazine and temozolomide, being prodrugs, ultimately yield MTIC as their active metabolite within the human body (21, 22). This shared metabolic fate underscores a commonality in their pharmacological profiles. Consequently, both agents have been observed to engage in a biochemical interaction with the MGMT enzyme. This interaction has been substantiated by clinical observations in patients with metastatic colorectal cancer, where a higher objective response rate to these alkylating agents has been correlated with the epigenetic silencing of the MGMT gene, an alteration that diminishes the DNA repair capacity of cancer cells, thereby enhancing the cytotoxic efficacy of the treatment (23, 24).

### Streptozocin

**Streptozocin**, a glucosamine-nitrosourea derivative, functions as an alkylating agent that impedes DNA synthesis through the processes of alkylation and DNA strand cross-linking. This compound is clinically indicated for the treatment of adrenocortical carcinoma and neuroendocrine tumors (NETs). Emerging evidence from a randomized phase II clinical trial suggests that the hypermethylation status of MGMT, denoted as hypermethylated MGMT (hmMGMT), may serve as a predictive biomarker for the therapeutic response to alkylating agents. The literature indicates that patients characterized by hmMGMT phenotypes demonstrate superior objective response rates, extended durations of progression-free survival, and enhanced overall survival outcomes when managed with streptozotocin, dacarbazine, or temozolomide. This contrasts with the outcomes observed in patients treated with oxaliplatin who also exhibit the hmMGMT phenotype. These findings underscore the potential of hmMGMT as a stratifying factor in the optimization of chemotherapeutic strategies, particularly in the context of alkylating agent selection for targeted cancer therapies (23).

## MGMT and other alkylators

### Nitrosoureas

*Nitrosoureas*, exemplified by carmustine, represent another class of alkylating agents utilized in the chemotherapy arsenal, with MGMT playing a pivotal role in ameliorating the genotoxic effects induced by these compounds. In the specific context of recurrent glioblastoma multiforme, a notable correlation has been observed between the hypermethylation status of the MGMT gene and a more favorable clinical outcome. Patients exhibiting this epigenetic modification have been reported to experience a heightened progression-free survival following the implantation of carmustine wafers, as well as an improved overall survival upon multivariate analysis, in stark contrast to those harboring the wild-type form of MGMT (24). The methylation status of the MGMT promoter in glioma patients has thus emerged as a valuable prognostic biomarker, offering a predictive insight into the tumor’s responsiveness to carmustine therapy (25).

Recent studies have highlighted the significance of lomustine, an alkylating agent, in the treatment of newly diagnosed MGMT methylated glioblastoma, despite previous assertions of limited data supporting its definitive interaction with MGMT. Notably, the combination of lomustine and temozolomide (TMZ) upfront presents a promising therapeutic strategy for this patient cohort. Schneider et al. (26) demonstrated that lomustine exhibits synergistic cytotoxic effects in MGMT promoter methylated glioblastoma cells, independent of MGMT status, suggesting a potential enhancement of treatment efficacy when used in conjunction with TMZ. Furthermore, a computational biological modeling study by Castro et al. (27) compared the outcomes of m-MGMT glioblastoma patients treated with either TMZ, lomustine, or a combination thereof, revealing that the combination therapy could potentially offer superior survival benefits.

Moreover, the CeTeG/NOA-09 trial, as discussed by Weller et al. (28), provides compelling evidence on the neurocognitive functioning benefits and improved survival rates associated with the lomustine-TMZ combination in patients with newly diagnosed, MGMT-methylated glioblastoma. This trial underscores the importance of considering lomustine in the treatment regimen for such patients. Additionally, the study by Fishman et al. (29) on Tumor Treating Fields (TTFields) further supports the use of lomustine by demonstrating its synergistic interaction with TTFields and TMZ in glioblastoma cell lines, regardless of MGMT expression levels.

### Alkyl sulfonates (melphalan, chlorambucil, busulfan, thiotepa)

Busulfan, a chemotherapeutic alkyl sulfonate, is utilized in the management of certain leukemia types. Theoretically, MGMT is postulated to contribute to chemoresistance by facilitating the repair of alkylated DNA lesions. However, empirical evidence suggests that MGMT activity does not significantly influence busulfan sensitivity. Studies conducted *in vitro*, wherein melanoma cells transfected with MGMT were incubated with various alkylating agents including melphalan, chlorambucil, busulfan, and thiotepa, demonstrated no discernible difference in cell survival post-treatment (30).

### Fluoropyrimidine/platinum

Theoretically, dMGMT may influence the overall response to combination therapies of antimetabolites/platinum and alkylating agents. However, the MGMT promoter methylation yielded no benefit from 5-fluorouracil or oxaliplatin-based regimens in colorectal cancer (31). *In vitro* MGMT-transfected melanoma cells yield no difference in survival after being incubated with cisplatin (30).

## Radiotherapy and MGMT

In glioblastoma patients undergoing radiotherapy as the sole post-resection treatment, the correlation between MGMT promoter methylation and enhanced response to radiotherapy was evident. The phenomenon where glioblastomas with an unmethylated MGMT promoter are twice as likely to exhibit disease progression during the course of radiation therapy underscores the significance of MGMT promoter methylation. These observations suggest that MGMT promoter methylation may serve as a promising predictive biomarker for gauging the responsiveness to radiotherapy. While the precise mechanisms that govern the interplay between MGMT activity and the effects of radiation therapy remain to be fully elucidated, it is hypothesized that these mechanisms may bear resemblance to the interactions observed with alkylating chemotherapeutic agents. This hypothesis is grounded in the understanding that both radiation and alkylating agents inflict damage upon the DNA, to which the MGMT enzyme is known to respond (32).

## Testing MGMT

The quantification of MGMT levels holds considerable prognostic value for anticipating the therapeutic efficacy of alkylating agents, thereby rendering MGMT assessment an integral aspect of the treatment planning process for certain cancers. A multitude of techniques has been developed to measure MGMT protein expression, including immunohistochemistry (33), and MGMT enzymatic activity assays, which can be conducted via *in situ* hybridization (34), among other methods.

Conversely, in scenarios where direct assessment of MGMT enzyme levels is compromised by factors such as limited sample availability, variability between laboratories, and challenges in result interpretation, complementary approaches that evaluate DNA methylation status gain clinical relevance. The literature documents several methodologies for this purpose, including real-time quantitative PCR (35), methylation-specific PCR (MS-PCR) (36), and pyrosequencing (23, 37). Notably, methylation-specific polymerase chain reaction analysis stands out as a high-throughput, quantitative methylation assay (24), offering a robust alternative for MGMT status determination in a clinical setting.

The Methylation-Sensitive Quantitative Locked Nucleic Acid PCR (MS-qLNA PCR) constitutes an advanced real-time PCR methodology. Distinguished from MS-PCR, this technique involves the amplification of the methylated and unmethylated MGMT gene promoter via PCR, with subsequent detection employing fluorophoric labeling. This approach significantly improves analytical sensitivity, thereby enabling the detection of minute tumoral cell populations and diminishing the incidence of false-negative outcomes (38).

Under certain conditions, the integration of two or more diagnostic techniques has been demonstrated to be necessary. For instance, the MGMT-NET randomized phase II trial utilized a combination of immunohistochemistry and pyrosequencing for methodological robustness (23).

NCCN endorses a variety of methodologies for assessing MGMT methylation status, including MS-PCR, high-resolution melting analysis specific to methylation, pyrosequencing, and droplet-digital PCR. One investigation posited that, among patients with GBM undergoing treatment with TMZ, pyrosequencing serves as the superior prognostic stratifier. Nonetheless, quantitative methylation-specific PCR (qMS-PCR) is the assay that has undergone the most extensive validation within the context of clinical trials.

## Strategies to overcome MGMT-mediated resistance

Investigators have been examining strategies to circumvent MGMT-mediated resistance, thereby augmenting the effectiveness of alkylating chemotherapeutic agents. In theory, if the activity of DNA alkylating agents exceeds the synthetic capacity of the MGMT enzyme, the enzyme becomes depleted, allowing the alkylating agent to maintain its therapeutic function. A decade prior, alternative dosing regimens were investigated, which included either an escalation of temozolomide dosage or an extension of the dosing period in patients with glioma (39). Presently, such strategies may be employed intermittently through off-label dosing in clinical practice; however, comprehensive assessments of their efficacy and safety remain to be ascertained.

Another avenue of research involves the deployment of MGMT inhibitors, which have the potential to sensitize cancer cells to the cytotoxic effects of agents such as temozolomide and dacarbazine. Clinical trials are in progress to determine the efficacy of integrating MGMT inhibitors with conventional chemotherapy to enhance patient outcomes. To date, no MGMT inhibitors have been sanctioned for clinical use. Nonetheless, early-stage research has yielded several strategies, including direct enzyme inhibition, MGMT gene silencing, RNA interference, poly (ADP-ribose) polymerase inhibition (PARPi), and the use of oncolytic viruses.

Compounds such as O(6)-benzylguanine (O(6)BG), 8-aza-O(6)-benzylguanine, and O(6)-(4-bromothenyl)guanine (O(6)BTG, also known as lomeguatrib) have been scrutinized for their capacity to inhibit the MGMT enzyme over the past decades. These molecules act as pseudosubstrates that engage in an enzyme-substrate interaction, which ultimately results in the enzyme’s inactivation. Phase II clinical trials assessing lomeguatrib in patients with melanoma have demonstrated that lomeguatrib reduces MGMT activity and potentiates the efficacy of temozolomide (40). Furthermore, lomeguatrib has been proposed as a potential radiosensitizer for glioblastoma cells with unmethylated MGMT (41). The conjugation of these inhibitory agents to glucose or folate esters has also been explored, with the rationale that cancer cells exhibit high levels of glucose or folate transporters, potentially facilitating enhanced transmembrane permeation. Studies have indicated that such conjugations may amplify the therapeutic impact of temozolomide (42, 43).

The zinc-porphyrin complex, Zn-DIGPor, demonstrates an affinity for binding to MGMT DNA through O(6)-methylguanine (O(6)-MeG) and attenuates MGMT expression at the genetic level. The addition of Zn-DIGPor has been shown to synergistically enhance the efficacy of temozolomide in cell lines expressing MGMT (44). Additional preliminary interventions targeting DNA have been investigated, including RNA interference techniques and the use of genetically engineered oncolytic viruses.

Furthermore, the attenuation of sensitivity caused by MGMT may be counteracted by the addition of olaparib. Both *in vitro* and *in vivo* studies indicate that cancer cells with MGMT expression exhibit a pronounced response to the combined administration of temozolomide and PARPi. Specifically, in MGMT-positive melanoma cells, the antiproliferative impact of temozolomide is substantially augmented when used in conjunction with PARPi, compared to the administration of temozolomide as a monotherapy. Nevertheless, the precise nature of this synergistic effect—whether it stems from direct inhibition of the MGMT enzyme or from the disruption of alternative DNA repair pathways—remains to be elucidated. Without additional foundational research, it is premature to assert that PARPi can inhibit MGMT (45). PARP inhibitors may introduce novel poly(ADP-ribosyl)ation modifications to MGMT, thereby neutralizing its function and enhancing the susceptibility of MGMT wild-type glioblastoma to temozolomide-induced cytotoxicity (46).

Resistance to TMZ has been observed in tumor cells that exhibit deficiencies in the DNA mismatch repair (MMR) system or the base excision repair (BER) system. Notably, this resistance mechanism operates independently of MGMT levels. The development of methods to counteract resistance stemming from these repair systems remains an area of uncertainty and necessitates further research.

Despite this, the momentum in developing strategies to augment the sensitivity of alkylating agents has waned as the oncology field has pivoted towards emerging modalities such as immunotherapy, antibody-drug conjugates, effector cell therapy, and bispecific antibodies. This transition coincided with the observation of increased toxicity when alkylators were used in combination with alternative oral sensitizers, and the challenges associated with demonstrating improved outcomes in overall survival.

One promising approach involves the targeted modulation of MGMT to enhance the efficacy of chemotherapy. Liu and Gerson (47) highlighted the clinical implications of MGMT modulation, emphasizing its potential to improve responses to alkylating agents in cancer therapy. Furthermore, Shaw et al. (48) discussed the multifaceted insights into MGMT-mediated resistance against TMZ and outlined the clinical trial perspectives that aim to address this resistance mechanism. These studies underscore the importance of understanding the complexities of MGMT-mediated resistance and the need for continued research in this area.

Additionally, the use of nanotechnology to target MGMT-mediated resistance in glioblastoma represents an innovative strategy. Torres et al. (49) reviewed the problems of MGMT inhibitors in clinical trials, suggesting that the nano-based systems could be effective vehicles for overcoming systemic toxicity in GBM patients. MGMT inhibitors could be delivered accumulatively to local tumor tissues, but minimally to healthy tissues. This approach exemplifies the ongoing efforts to develop targeted delivery that can bypass traditional resistance mechanisms and improve treatment efficacy.

## Conclusion

It becomes increasingly apparent that elucidating the complex functions and regulatory pathways of MGMT is key to the development of more sophisticated and precise cancer therapies. By delving deeper into the molecular underpinnings of MGMT, researchers may unlock new possibilities for the creation of targeted treatment regimens that can more effectively combat the multifaceted challenges posed by cancer. The potential to tailor treatments based on the MGMT status of tumors offers a promising avenue for increasing the specificity and potency of cancer therapies, thereby improving the prognosis and quality of life for patients afflicted with this disease.

In conclusion, the enzyme MGMT stands as a critical determinant at the nexus of DNA repair and oncological pharmacotherapy, influencing the therapeutic success of alkylating agents such as temozolomide and dacarbazine. The intricate interplay between MGMT and these chemotherapeutic agents is of paramount importance for the development of strategies to circumvent resistance and enhance treatment efficacy in the realm of cancer therapy. As the body of research in this area expands, the unraveling of MGMT’s complexities may herald a new era of targeted and efficacious cancer treatments, offering hope for improved patient outcomes.

## Author contributions

LC: Conceptualization, Writing – original draft. AW: Conceptualization, Supervision, Writing – review & editing.
